# *catena*-Poly[[copper(I)-μ-2,6-bis­[4-(pyridin-2-yl)thia­zol-2-yl]pyridine] hexa­fluoridophosphate acetonitrile monosolvate] from single-crystal synchrotron data

**DOI:** 10.1107/S1600536813006831

**Published:** 2013-03-28

**Authors:** Linda Xiao, Mohan Bhadbhade, Anthony T. Baker

**Affiliations:** a School of Chemistry and Forensic Science, University of Technology, Sydney, PO Box 123, Broadway NSW 2007, Australia; b Mark Wainwright Analytical Centre, University of New South Wales, Anzac Parade, Sydney, New South Wales, 2052, Australia

## Abstract

The title complex, {[Cu(C_21_H_13_N_5_S_2_)]PF_6_·CH_3_CN}_*n*_, was formed immediately on adding together a methanol solution containing copper(I) ions and a methanol solution of 2,6-bis­[4-(pyridin-2-yl)thia­zol-2-yl]pyridine. Crystallographic studies of the complex reveal a coordination polymer with the ligand acting as a bis­(bidentate) ligand with the pyridine N atom not coordinating a metal centre. The Cu^I^ atom is four-coordinate with approximately tetra­hedral stereochemistry: the N_4_ donor set is provided by bipyridine-like moieties of the two heterocyclic ligands. Parallel chains of the coordination polymer run along the *b*-axis direction with the disordered (0.50:0.50 occupancy ratio) PF_6_^−^ anions and acetonitrile solvent mol­ecules located between the chains.

## Related literature

For a related complex, see: Baker & Matthews (1999 ▶).
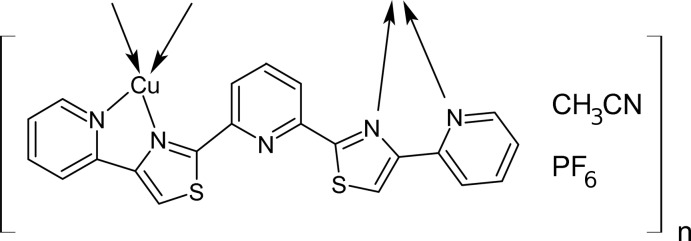


## Experimental

### 

#### Crystal data

[Cu(C_21_H_13_N_5_S_2_)]PF_6_·C_2_H_3_N
*M* *_r_* = 649.05
Monoclinic, 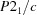

*a* = 12.525 (3) Å
*b* = 13.950 (3) Å
*c* = 14.626 (3) Å
β = 97.72 (3)°
*V* = 2532.4 (9) Å^3^
*Z* = 4
Synchrotron radiation
λ = 0.71073 Å
μ = 1.16 mm^−1^
*T* = 100 K
0.03 × 0.02 × 0.01 mm

#### Data collection

3-BM1 Australian Synchrotron diffractometer
28022 measured reflections
3890 independent reflections
3641 reflections with *I* > 2σ(*I*)
*R*_int_ = 0.024
θ_max_ = 23.8°

#### Refinement

*R*[*F* ^2^ > 2σ(*F* ^2^)] = 0.031
*wR*(*F* ^2^) = 0.078
*S* = 1.08
3890 reflections
389 parameters
H-atom parameters constrained
Δρ_max_ = 0.30 e Å^−3^
Δρ_min_ = −0.57 e Å^−3^

### 

Data collection: *BLU-ICE* (McPhillips *et al.*, 2002 ▶); cell refinement: *XDS* (Kabsch, 1993 ▶); data reduction: *XDS*; program(s) used to solve structure: *SHELXS97* (Sheldrick, 2008 ▶); program(s) used to refine structure: *SHELXL97* (Sheldrick, 2008 ▶); molecular graphics: *Mercury* (Macrae *et al.*, 2008 ▶); software used to prepare material for publication: *publCIF* (Westrip, 2010 ▶).

## Supplementary Material

Crystal structure: contains datablock(s) I, global. DOI: 10.1107/S1600536813006831/vm2190sup1.cif

Structure factors: contains datablock(s) I. DOI: 10.1107/S1600536813006831/vm2190Isup2.hkl

Additional supplementary materials:  crystallographic information; 3D view; checkCIF report

## Figures and Tables

**Fig. 1.**
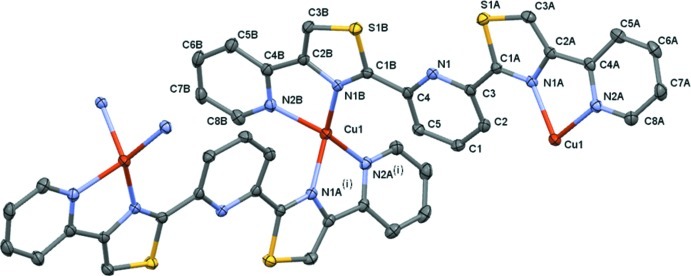
Thermal ellipsoids plot (40% probability) of the part of the coordination polymer showing the geometry around Cu(I) ion. Hydrogen atoms, the PF6 anion and the solvent acetonitrile molecule are omitted for clarity. Symmetry code: (i) -x + 1, y + 1/2, -z + 1/2.

**Fig. 2.**
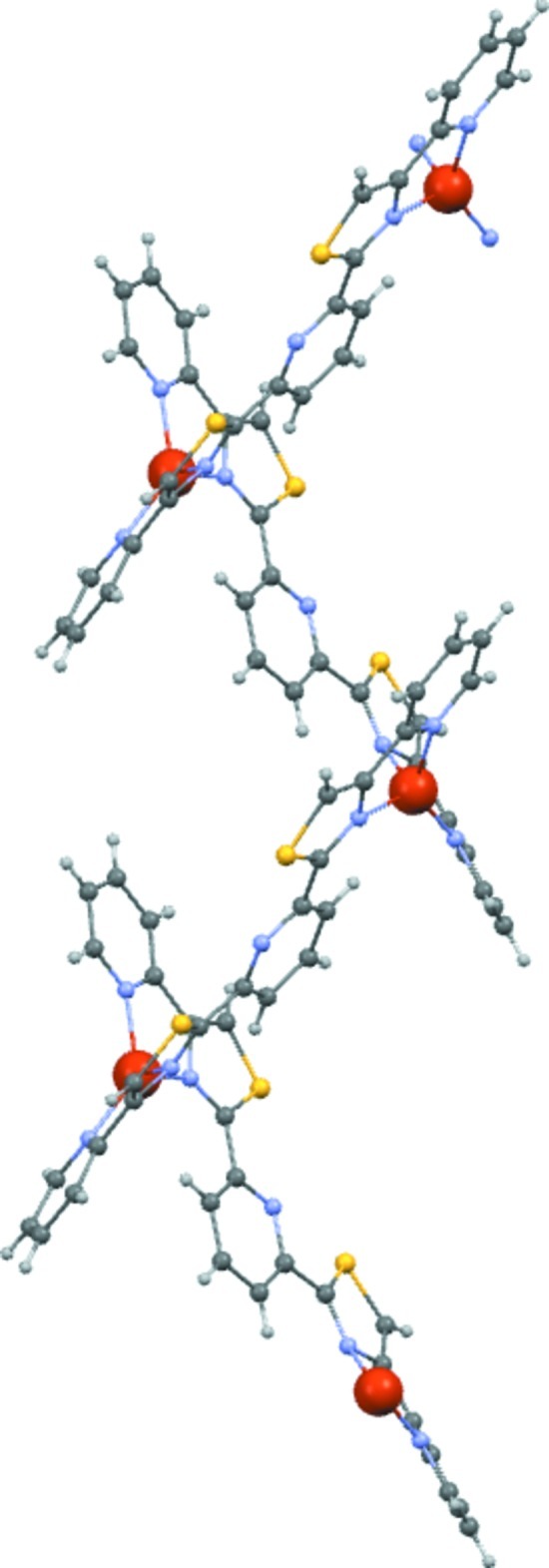
A single chain showing the construction of the coordination polymer formed with the ligand.

**Fig. 3.**
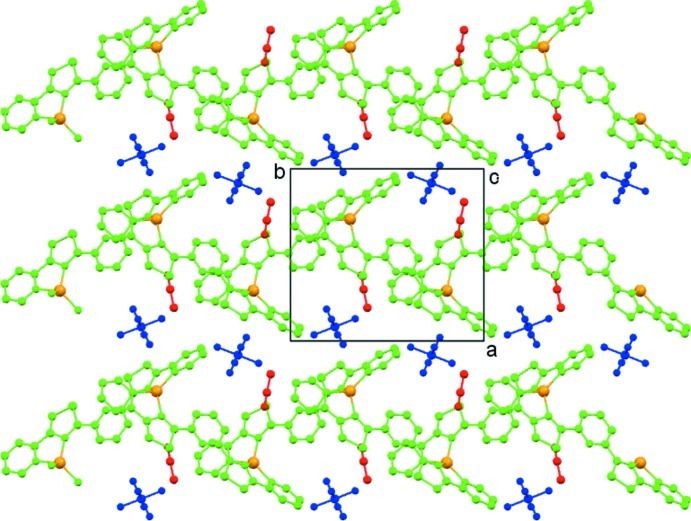
Packing of coordination polymers viewed down c axis that includes PF6 anions (disorder omitted for clarity) and solvent molecules (acetonitrile).

## supplementary crystallographic information

### Comment

We have prepared and studied many analogues of 2,2'-bipyridine and 2,2':6',2"-terpyridine (Baker and Matthews, 1999 and references therein) and have extended this work to the preparation of ligands analogous to quinquepyridine. For metal complexes of these quinquepyridine analogues, a number of features have been observed. The interpolation of a five-membered heterocycle appears to reduce the capacity of the ligands to employ all five donor atoms and we have seen several examples where the ligands act in a bis(bidentate) mode [2 + 2]. In such cases the ligands bridge between metal centres in binuclear complexes. Herein we report a coordination polymer shown in Scheme 1, again where the ligand binds in [2 + 2] mode. A thermal ellipsoid plot is shown in Fig. 1. Each copper centre has approximately tetrahedral stereochemistry as shown in Fig. 1. The principal cause of distortion being the bite angles of the bidentate ligand N2B—Cu1—N1B (82.47 (8)°) and N2A^i^—Cu1—N1A^i^ (82.95 (8)°) (symmetry code: (i) -*x* + 1, *y* + 1/2, -*z* + 1/2) are considerably less than the ideal tetrahedral angle. Two 'thiazolylpyridine' moieties coordinate each copper(I) centre with the relevant bond lengths being Cu—N1A^i^ 2.098 (2) Å, Cu—N1B 2.050 (2) Å, Cu—N2A 1.992 (2) Å and Cu—N2B 2.024 (2) Å. The Cu—N bond lengths are similar but the Cu—N_pyridinyl_ bonds are slightly shorter than the Cu—N_thiazolyl_ bonds. This indicates a slightly stronger interaction of the metal atom with the pyridinyl moiety, in line with base strength. A single chain of the coordination polymer, thus created, is depicted in Fig. 2 and packing of these chains that include PF_6_^-^ anions and solvent molecules of acetonitriles are shown in Fig. 3.

### Experimental

The quinquedentate ligand 2,6-bis(4-(pyridin-2-yl)thiazol-2-yl)pyridine was prepared by adding a solution of 2-(bromoacetyl)pyridinium hydrobromide (5.6 g, 20 mmol) in hot ethanol (50 ml) to a solution of 2,6-di(thioamido)pyridine (2.0 g, 10 mmol) in hot ethanol (50 ml). The solution was heated for 5 min, a yellow precipitate of 2,6-bis(4-(pyridin-2-yl)thiazol-2-yl)pyridinium hydrobromide separated soon. The mixture was allowed to stand for 30 min s and the yellow precipitate was filtered and washed with sodium bicarbonate (5%) until effervescence ceased. Yield: 75%. The complex was prepared as follows: Tetrakis(acetonitrile)copper(I) hexafluorophosphate (200 mg, 0.54 mmol) in hot methanol (20 ml) was added to a solution of the ligand (214 mg, 0.54 mmol) in hot methanol (20 ml). The reaction mixture was heated on the water bath for 1 h. An orange solid formed during this time and once cooled the solid was collected, washed with cooled methanol and stored over silica gel (yield 164 mg, 50%). Crystals were grown by vapour diffusion of diethyl ether into a concentrated acetonitrile solution of the complex.

### Refinement

All the H-atoms were fixed stereochemically and included in the refinement using riding model option in *SHELXL97*. The PF6 anion was found to exhibit orientational disorder, which was modelled over two positions.

H atoms were positioned geometrically with C—H = 0.93 - 0.96 Å. *U*_iso_(H) values were set at 1.2U_eq_ (aromatic) or 1.5U_eq_ of the parent atom (methyl group).

### Crystal data

**Table:**

[Cu(C_21_H_13_N_5_S_2_)]PF_6_·C_2_H_3_N	*F*(000) = 1304
*M**_r_* = 649.05	*D*_x_ = 1.702 Mg m^−^^3^
Monoclinic, *P*2_1_/*c*	Synchrotron radiation, λ = 0.71073 Å
Hall symbol: -P 2ybc	Cell parameters from 9980 reflections
*a* = 12.525 (3) Å	θ = 2.5–22.5°
*b* = 13.950 (3) Å	µ = 1.16 mm^−^^1^
*c* = 14.626 (3) Å	*T* = 100 K
β = 97.72 (3)°	Thin plates, blue
*V* = 2532.4 (9) Å^3^	0.03 × 0.02 × 0.01 mm
*Z* = 4	

### Data collection

**Table:**

3-BM1 Australian Synchrotron diffractometer	3641 reflections with *I* > 2σ(*I*)
Radiation source: Synchrotron BM	*R*_int_ = 0.024
Si<111> monochromator	θ_max_ = 23.8°, θ_min_ = 1.6°
φ scans	*h* = −14→14
28022 measured reflections	*k* = −15→15
3890 independent reflections	*l* = −16→16

### Refinement

**Table:**

Refinement on *F*^2^	Primary atom site location: structure-invariant direct methods
Least-squares matrix: full	Secondary atom site location: difference Fourier map
*R*[*F*^2^ > 2σ(*F*^2^)] = 0.031	Hydrogen site location: inferred from neighbouring sites
*wR*(*F*^2^) = 0.078	H-atom parameters constrained
*S* = 1.08	*w* = 1/[σ^2^(*F*_o_^2^) + (0.0359*P*)^2^ + 3.8973*P*],*P* = (*F*_o_^2^ + 2*F*_c_^2^)/3
3890 reflections	(Δ/σ)_max_ = 0.001
389 parameters	Δρ_max_ = 0.30 e Å^−^^3^
0 restraints	Δρ_min_ = −0.57 e Å^−^^3^

### Special details

**Table:**

Geometry. All e.s.d.'s (except the e.s.d. in the dihedral angle between two l.s. planes) are estimated using the full covariance matrix. The cell e.s.d.'s are taken into account individually in the estimation of e.s.d.'s in distances, angles and torsion angles; correlations between e.s.d.'s in cell parameters are only used when they are defined by crystal symmetry. An approximate (isotropic) treatment of cell e.s.d.'s is used for estimating e.s.d.'s involving l.s. planes.
Refinement. Refinement of *F*^2^ against ALL reflections. The weighted *R*-factor *wR* and goodness of fit *S* are based on *F*^2^, conventional *R*-factors *R* are based on *F*, with *F* set to zero for negative *F*^2^. The threshold expression of *F*^2^ > σ(*F*^2^) is used only for calculating *R*-factors(gt) *etc*. and is not relevant to the choice of reflections for refinement. *R*-factors based on *F*^2^ are statistically about twice as large as those based on *F*, and *R*- factors based on ALL data will be even larger.

### Fractional atomic coordinates and isotropic or equivalent isotropic displacement parameters (Å^2^)

**Table:**

	*x*	*y*	*z*	*U*_iso_*/*U*_eq_	Occ. (<1)
Cu1	0.70815 (2)	1.19154 (2)	0.36895 (2)	0.01965 (11)	
N1	0.40269 (16)	0.95445 (14)	0.35587 (13)	0.0159 (4)	
C1	0.5696 (2)	0.89759 (19)	0.25992 (17)	0.0211 (6)	
H1	0.6257	0.8784	0.2285	0.025*	
C2	0.4814 (2)	0.83892 (18)	0.26281 (17)	0.0197 (5)	
H2	0.4773	0.7794	0.2339	0.024*	
C3	0.3990 (2)	0.87086 (17)	0.30999 (16)	0.0165 (5)	
C4	0.48924 (19)	1.00992 (17)	0.35293 (16)	0.0162 (5)	
C5	0.5735 (2)	0.98545 (18)	0.30444 (17)	0.0197 (5)	
H5	0.6311	1.0270	0.3020	0.024*	
S1A	0.21354 (5)	0.84000 (5)	0.39190 (4)	0.02002 (16)	
N1A	0.27744 (16)	0.73513 (14)	0.26615 (14)	0.0163 (4)	
N2A	0.17898 (16)	0.59744 (15)	0.15433 (14)	0.0180 (4)	
C1A	0.3024 (2)	0.81261 (17)	0.31486 (16)	0.0160 (5)	
C2A	0.18499 (19)	0.69316 (17)	0.28980 (17)	0.0173 (5)	
C3A	0.1400 (2)	0.74016 (19)	0.35688 (17)	0.0207 (5)	
H3A	0.0781	0.7207	0.3803	0.025*	
C4A	0.14469 (19)	0.60735 (18)	0.23785 (17)	0.0173 (5)	
C5A	0.0784 (2)	0.5404 (2)	0.27187 (18)	0.0239 (6)	
H5A	0.0574	0.5484	0.3300	0.029*	
C6A	0.0438 (2)	0.4617 (2)	0.21859 (19)	0.0276 (6)	
H6A	−0.0001	0.4157	0.2405	0.033*	
C7A	0.0757 (2)	0.4527 (2)	0.13181 (19)	0.0275 (6)	
H7A	0.0520	0.4015	0.0937	0.033*	
C8A	0.1432 (2)	0.52119 (18)	0.10326 (18)	0.0232 (6)	
H8A	0.1652	0.5142	0.0454	0.028*	
S1B	0.37811 (5)	1.12599 (5)	0.46088 (4)	0.02089 (16)	
N1B	0.56301 (16)	1.16578 (14)	0.41446 (13)	0.0154 (4)	
N2B	0.69867 (17)	1.31538 (14)	0.43946 (14)	0.0193 (5)	
C1B	0.48752 (19)	1.09985 (18)	0.40534 (16)	0.0165 (5)	
C2B	0.5353 (2)	1.24125 (18)	0.46810 (16)	0.0173 (5)	
C3B	0.4376 (2)	1.23154 (18)	0.49859 (17)	0.0207 (5)	
H4B	0.4076	1.2762	0.5349	0.025*	
C4B	0.6105 (2)	1.32303 (18)	0.48297 (17)	0.0190 (5)	
C5B	0.5930 (2)	1.40191 (19)	0.53706 (18)	0.0248 (6)	
H5B	0.5330	1.4047	0.5682	0.030*	
C6B	0.6670 (2)	1.47623 (19)	0.54353 (19)	0.0290 (6)	
H6B	0.6566	1.5303	0.5785	0.035*	
C7B	0.7559 (2)	1.46972 (19)	0.49804 (18)	0.0263 (6)	
H7B	0.8059	1.5193	0.5013	0.032*	
C8B	0.7696 (2)	1.38795 (19)	0.44740 (18)	0.0239 (6)	
H8B	0.8305	1.3831	0.4176	0.029*	
P1	0.91781 (5)	0.77051 (5)	0.06197 (5)	0.02250 (17)	
F1	1.02028 (15)	0.72763 (13)	0.02232 (14)	0.0467 (5)	
F2	0.9637 (5)	0.7561 (5)	0.1692 (5)	0.0320 (13)	0.50
F3	0.8706 (11)	0.6650 (9)	0.0595 (7)	0.034 (2)	0.50
F4	0.8738 (5)	0.7876 (5)	−0.0432 (4)	0.0503 (15)	0.50
F5	0.9640 (8)	0.8778 (6)	0.0694 (4)	0.0348 (15)	0.50
F2'	1.0012 (6)	0.7796 (6)	0.1511 (5)	0.064 (2)	0.50
F3'	0.8878 (13)	0.6654 (11)	0.0896 (8)	0.063 (4)	0.50
F4'	0.8345 (5)	0.7575 (6)	−0.0299 (5)	0.063 (2)	0.50
F5'	0.9533 (8)	0.8721 (7)	0.0272 (6)	0.080 (3)	0.50
F6	0.81523 (16)	0.81302 (15)	0.10063 (17)	0.0572 (6)	
C1AN	0.1934 (3)	0.1022 (3)	0.2238 (2)	0.0507 (9)	
H1A1	0.1468	0.0772	0.1718	0.076*	
H1A2	0.1637	0.1606	0.2442	0.076*	
H1A3	0.2001	0.0562	0.2730	0.076*	
C2AN	0.2998 (3)	0.1214 (2)	0.19694 (19)	0.0297 (7)	
N1AN	0.3824 (2)	0.13309 (19)	0.17597 (18)	0.0368 (6)	

### Atomic displacement parameters (Å^2^)

**Table:**

	*U*^11^	*U*^22^	*U*^33^	*U*^12^	*U*^13^	*U*^23^
Cu1	0.02253 (19)	0.01556 (18)	0.02245 (18)	0.00197 (12)	0.00884 (13)	−0.00093 (12)
N1	0.0184 (11)	0.0143 (10)	0.0151 (10)	0.0000 (8)	0.0026 (8)	0.0017 (8)
C1	0.0218 (13)	0.0219 (14)	0.0209 (13)	0.0003 (11)	0.0083 (10)	−0.0021 (11)
C2	0.0243 (14)	0.0154 (13)	0.0195 (13)	−0.0016 (10)	0.0031 (10)	−0.0016 (10)
C3	0.0198 (13)	0.0146 (12)	0.0146 (12)	0.0000 (10)	0.0012 (10)	0.0032 (10)
C4	0.0214 (13)	0.0130 (12)	0.0137 (12)	−0.0013 (10)	0.0000 (10)	0.0016 (10)
C5	0.0195 (13)	0.0194 (13)	0.0210 (13)	−0.0038 (10)	0.0058 (10)	0.0006 (10)
S1A	0.0198 (3)	0.0213 (3)	0.0196 (3)	0.0006 (3)	0.0054 (2)	−0.0028 (3)
N1A	0.0173 (10)	0.0145 (11)	0.0172 (10)	0.0002 (8)	0.0024 (8)	0.0026 (9)
N2A	0.0174 (10)	0.0165 (11)	0.0199 (11)	0.0017 (9)	0.0022 (8)	0.0025 (9)
C1A	0.0179 (12)	0.0151 (13)	0.0150 (12)	0.0023 (10)	0.0025 (10)	0.0023 (10)
C2A	0.0157 (12)	0.0173 (13)	0.0188 (12)	0.0003 (10)	0.0016 (10)	0.0047 (10)
C3A	0.0173 (13)	0.0243 (14)	0.0211 (13)	−0.0019 (11)	0.0046 (10)	0.0023 (11)
C4A	0.0148 (12)	0.0160 (13)	0.0207 (13)	0.0019 (10)	0.0009 (10)	0.0041 (10)
C5A	0.0221 (14)	0.0262 (14)	0.0233 (13)	−0.0032 (11)	0.0029 (11)	0.0052 (11)
C6A	0.0258 (14)	0.0227 (14)	0.0337 (15)	−0.0093 (11)	0.0023 (12)	0.0069 (12)
C7A	0.0317 (15)	0.0188 (14)	0.0310 (15)	−0.0073 (12)	0.0004 (12)	−0.0022 (12)
C8A	0.0272 (14)	0.0199 (14)	0.0226 (13)	−0.0005 (11)	0.0036 (11)	−0.0002 (11)
S1B	0.0197 (3)	0.0201 (3)	0.0244 (3)	−0.0023 (3)	0.0085 (3)	−0.0034 (3)
N1B	0.0189 (11)	0.0132 (10)	0.0146 (10)	0.0000 (8)	0.0038 (8)	0.0009 (8)
N2B	0.0225 (11)	0.0152 (11)	0.0197 (11)	0.0005 (9)	0.0010 (9)	0.0010 (9)
C1B	0.0181 (12)	0.0178 (13)	0.0141 (12)	0.0018 (10)	0.0039 (10)	0.0021 (10)
C2B	0.0230 (13)	0.0148 (12)	0.0142 (12)	0.0020 (10)	0.0025 (10)	0.0001 (10)
C3B	0.0231 (14)	0.0186 (13)	0.0212 (13)	0.0005 (11)	0.0059 (10)	−0.0051 (11)
C4B	0.0229 (13)	0.0166 (13)	0.0172 (12)	0.0019 (10)	0.0013 (10)	0.0019 (10)
C5B	0.0294 (15)	0.0196 (14)	0.0253 (14)	0.0035 (11)	0.0037 (11)	−0.0033 (11)
C6B	0.0404 (17)	0.0173 (14)	0.0288 (15)	0.0022 (12)	0.0022 (13)	−0.0060 (11)
C7B	0.0336 (16)	0.0153 (13)	0.0283 (15)	−0.0057 (11)	−0.0021 (12)	−0.0008 (11)
C8B	0.0236 (14)	0.0212 (14)	0.0269 (14)	−0.0031 (11)	0.0030 (11)	0.0038 (11)
P1	0.0214 (4)	0.0218 (4)	0.0244 (4)	0.0027 (3)	0.0035 (3)	0.0035 (3)
F1	0.0402 (11)	0.0389 (11)	0.0688 (13)	0.0088 (8)	0.0354 (10)	0.0116 (9)
F2	0.046 (4)	0.029 (2)	0.021 (2)	0.009 (2)	0.003 (2)	0.0040 (16)
F3	0.033 (4)	0.020 (4)	0.051 (5)	−0.010 (3)	0.016 (3)	−0.016 (4)
F4	0.064 (5)	0.061 (4)	0.023 (2)	0.018 (3)	−0.008 (3)	0.011 (2)
F5	0.046 (3)	0.017 (2)	0.046 (3)	−0.001 (2)	0.024 (3)	−0.002 (3)
F2'	0.053 (5)	0.092 (6)	0.039 (4)	0.035 (4)	−0.018 (3)	−0.038 (4)
F3'	0.051 (6)	0.043 (5)	0.103 (10)	0.004 (4)	0.037 (6)	0.032 (6)
F4'	0.031 (3)	0.113 (6)	0.041 (3)	0.001 (3)	−0.010 (2)	0.001 (3)
F5'	0.038 (3)	0.030 (4)	0.176 (9)	0.006 (3)	0.027 (6)	0.054 (6)
F6	0.0392 (11)	0.0510 (13)	0.0864 (16)	0.0157 (9)	0.0267 (11)	−0.0079 (11)
C1AN	0.0362 (19)	0.077 (3)	0.0421 (19)	−0.0020 (18)	0.0159 (15)	0.0064 (19)
C2AN	0.0368 (18)	0.0305 (16)	0.0215 (14)	0.0006 (13)	0.0026 (13)	0.0061 (12)
N1AN	0.0347 (16)	0.0418 (16)	0.0340 (14)	−0.0042 (12)	0.0049 (12)	0.0149 (12)

### Geometric parameters (Å, º)

**Table:**

Cu1—N2A^i^	1.992 (2)	C8A—H8A	0.9300
Cu1—N2B	2.024 (2)	S1B—C3B	1.708 (3)
Cu1—N1B	2.050 (2)	S1B—C1B	1.723 (2)
Cu1—N1A^i^	2.098 (2)	N1B—C1B	1.313 (3)
N1—C4	1.337 (3)	N1B—C2B	1.385 (3)
N1—C3	1.343 (3)	N2B—C8B	1.342 (3)
C1—C2	1.380 (4)	N2B—C4B	1.351 (3)
C1—C5	1.386 (4)	C2B—C3B	1.364 (4)
C1—H1	0.9300	C2B—C4B	1.476 (4)
C2—C3	1.390 (4)	C3B—H4B	0.9300
C2—H2	0.9300	C4B—C5B	1.390 (4)
C3—C1A	1.467 (3)	C5B—C6B	1.385 (4)
C4—C5	1.391 (4)	C5B—H5B	0.9300
C4—C1B	1.472 (3)	C6B—C7B	1.375 (4)
C5—H5	0.9300	C6B—H6B	0.9300
S1A—C3A	1.710 (3)	C7B—C8B	1.383 (4)
S1A—C1A	1.730 (2)	C7B—H7B	0.9300
N1A—C1A	1.309 (3)	C8B—H8B	0.9300
N1A—C2A	1.382 (3)	P1—F2'	1.562 (7)
N1A—Cu1^ii^	2.098 (2)	P1—F3'	1.580 (16)
N2A—C8A	1.342 (3)	P1—F4	1.581 (6)
N2A—C4A	1.355 (3)	P1—F3	1.585 (13)
N2A—Cu1^ii^	1.992 (2)	P1—F6	1.587 (2)
C2A—C3A	1.363 (4)	P1—F5'	1.589 (9)
C2A—C4A	1.470 (4)	P1—F1	1.5941 (18)
C3A—H3A	0.9300	P1—F4'	1.597 (7)
C4A—C5A	1.385 (4)	P1—F5	1.603 (9)
C5A—C6A	1.382 (4)	P1—F2	1.609 (7)
C5A—H5A	0.9300	C1AN—C2AN	1.464 (4)
C6A—C7A	1.387 (4)	C1AN—H1A1	0.9600
C6A—H6A	0.9300	C1AN—H1A2	0.9600
C7A—C8A	1.377 (4)	C1AN—H1A3	0.9600
C7A—H7A	0.9300	C2AN—N1AN	1.130 (4)
			
N2A^i^—Cu1—N2B	137.85 (9)	S1B—C3B—H4B	124.7
N2A^i^—Cu1—N1B	128.37 (8)	N2B—C4B—C5B	122.1 (2)
N2B—Cu1—N1B	82.47 (8)	N2B—C4B—C2B	114.5 (2)
N2A^i^—Cu1—N1A^i^	82.95 (8)	C5B—C4B—C2B	123.3 (2)
N2B—Cu1—N1A^i^	104.53 (8)	C6B—C5B—C4B	118.5 (3)
N1B—Cu1—N1A^i^	123.28 (8)	C6B—C5B—H5B	120.8
C4—N1—C3	117.4 (2)	C4B—C5B—H5B	120.8
C2—C1—C5	119.2 (2)	C7B—C6B—C5B	119.7 (3)
C2—C1—H1	120.4	C7B—C6B—H6B	120.1
C5—C1—H1	120.4	C5B—C6B—H6B	120.1
C1—C2—C3	118.4 (2)	C6B—C7B—C8B	118.7 (3)
C1—C2—H2	120.8	C6B—C7B—H7B	120.7
C3—C2—H2	120.8	C8B—C7B—H7B	120.7
N1—C3—C2	123.3 (2)	N2B—C8B—C7B	122.7 (3)
N1—C3—C1A	115.5 (2)	N2B—C8B—H8B	118.6
C2—C3—C1A	121.3 (2)	C7B—C8B—H8B	118.6
N1—C4—C5	123.3 (2)	F2'—P1—F3'	91.0 (5)
N1—C4—C1B	114.1 (2)	F2'—P1—F4	155.0 (4)
C5—C4—C1B	122.7 (2)	F3'—P1—F4	109.0 (5)
C1—C5—C4	118.4 (2)	F2'—P1—F3	107.4 (5)
C1—C5—H5	120.8	F3'—P1—F3	16.8 (6)
C4—C5—H5	120.8	F4—P1—F3	92.2 (5)
C3A—S1A—C1A	89.57 (12)	F2'—P1—F6	98.9 (3)
C1A—N1A—C2A	111.1 (2)	F3'—P1—F6	91.4 (6)
C1A—N1A—Cu1^ii^	135.01 (17)	F4—P1—F6	95.6 (3)
C2A—N1A—Cu1^ii^	107.09 (15)	F3—P1—F6	92.2 (5)
C8A—N2A—C4A	117.4 (2)	F2'—P1—F5'	90.6 (5)
C8A—N2A—Cu1^ii^	128.23 (18)	F3'—P1—F5'	174.9 (7)
C4A—N2A—Cu1^ii^	113.89 (16)	F4—P1—F5'	68.3 (4)
N1A—C1A—C3	124.7 (2)	F3—P1—F5'	160.2 (5)
N1A—C1A—S1A	114.16 (18)	F6—P1—F5'	93.2 (3)
C3—C1A—S1A	121.09 (18)	F2'—P1—F1	81.6 (3)
C3A—C2A—N1A	114.7 (2)	F3'—P1—F1	88.6 (6)
C3A—C2A—C4A	128.1 (2)	F4—P1—F1	84.0 (3)
N1A—C2A—C4A	117.2 (2)	F3—P1—F1	87.7 (5)
C2A—C3A—S1A	110.47 (19)	F6—P1—F1	179.52 (13)
C2A—C3A—H3A	124.8	F5'—P1—F1	86.8 (3)
S1A—C3A—H3A	124.8	F2'—P1—F4'	177.9 (4)
N2A—C4A—C5A	122.2 (2)	F3'—P1—F4'	87.6 (5)
N2A—C4A—C2A	114.9 (2)	F4—P1—F4'	25.2 (2)
C5A—C4A—C2A	122.9 (2)	F3—P1—F4'	71.1 (5)
C6A—C5A—C4A	119.4 (2)	F6—P1—F4'	82.8 (3)
C6A—C5A—H5A	120.3	F5'—P1—F4'	90.7 (4)
C4A—C5A—H5A	120.3	F1—P1—F4'	96.8 (3)
C5A—C6A—C7A	118.8 (2)	F2'—P1—F5	70.9 (4)
C5A—C6A—H6A	120.6	F3'—P1—F5	161.0 (4)
C7A—C6A—H6A	120.6	F4—P1—F5	90.0 (3)
C8A—C7A—C6A	118.6 (3)	F3—P1—F5	177.2 (5)
C8A—C7A—H7A	120.7	F6—P1—F5	86.0 (3)
C6A—C7A—H7A	120.7	F5'—P1—F5	22.4 (4)
N2A—C8A—C7A	123.6 (2)	F1—P1—F5	94.2 (3)
N2A—C8A—H8A	118.2	F4'—P1—F5	110.7 (4)
C7A—C8A—H8A	118.2	F2'—P1—F2	23.9 (3)
C3B—S1B—C1B	89.68 (12)	F3'—P1—F2	72.6 (4)
C1B—N1B—C2B	111.0 (2)	F4—P1—F2	178.4 (3)
C1B—N1B—Cu1	138.40 (17)	F3—P1—F2	89.4 (4)
C2B—N1B—Cu1	110.56 (16)	F6—P1—F2	84.2 (2)
C8B—N2B—C4B	118.3 (2)	F5'—P1—F2	110.2 (4)
C8B—N2B—Cu1	127.22 (18)	F1—P1—F2	96.2 (2)
C4B—N2B—Cu1	114.53 (17)	F4'—P1—F2	156.0 (3)
N1B—C1B—C4	126.1 (2)	F5—P1—F2	88.4 (3)
N1B—C1B—S1B	114.28 (18)	C2AN—C1AN—H1A1	109.5
C4—C1B—S1B	119.61 (18)	C2AN—C1AN—H1A2	109.5
C3B—C2B—N1B	114.4 (2)	H1A1—C1AN—H1A2	109.5
C3B—C2B—C4B	127.6 (2)	C2AN—C1AN—H1A3	109.5
N1B—C2B—C4B	117.9 (2)	H1A1—C1AN—H1A3	109.5
C2B—C3B—S1B	110.60 (19)	H1A2—C1AN—H1A3	109.5
C2B—C3B—H4B	124.7	N1AN—C2AN—C1AN	177.8 (4)
			
C5—C1—C2—C3	0.5 (4)	N2B—Cu1—N1B—C1B	176.7 (3)
C4—N1—C3—C2	2.0 (3)	N1A^i^—Cu1—N1B—C1B	74.2 (3)
C4—N1—C3—C1A	−179.8 (2)	N2A^i^—Cu1—N1B—C2B	147.28 (15)
C1—C2—C3—N1	−2.6 (4)	N2B—Cu1—N1B—C2B	−0.84 (16)
C1—C2—C3—C1A	179.3 (2)	N1A^i^—Cu1—N1B—C2B	−103.31 (16)
C3—N1—C4—C5	0.6 (3)	N2A^i^—Cu1—N2B—C8B	39.4 (3)
C3—N1—C4—C1B	−179.6 (2)	N1B—Cu1—N2B—C8B	−178.7 (2)
C2—C1—C5—C4	1.9 (4)	N1A^i^—Cu1—N2B—C8B	−56.2 (2)
N1—C4—C5—C1	−2.6 (4)	N2A^i^—Cu1—N2B—C4B	−140.17 (17)
C1B—C4—C5—C1	177.7 (2)	N1B—Cu1—N2B—C4B	1.73 (17)
C2A—N1A—C1A—C3	176.6 (2)	N1A^i^—Cu1—N2B—C4B	124.25 (17)
Cu1^ii^—N1A—C1A—C3	−37.1 (4)	C2B—N1B—C1B—C4	179.8 (2)
C2A—N1A—C1A—S1A	−0.7 (3)	Cu1—N1B—C1B—C4	2.3 (4)
Cu1^ii^—N1A—C1A—S1A	145.62 (16)	C2B—N1B—C1B—S1B	0.3 (3)
N1—C3—C1A—N1A	171.2 (2)	Cu1—N1B—C1B—S1B	−177.16 (14)
C2—C3—C1A—N1A	−10.5 (4)	N1—C4—C1B—N1B	179.0 (2)
N1—C3—C1A—S1A	−11.7 (3)	C5—C4—C1B—N1B	−1.2 (4)
C2—C3—C1A—S1A	166.63 (19)	N1—C4—C1B—S1B	−1.5 (3)
C3A—S1A—C1A—N1A	0.76 (19)	C5—C4—C1B—S1B	178.24 (19)
C3A—S1A—C1A—C3	−176.6 (2)	C3B—S1B—C1B—N1B	−0.1 (2)
C1A—N1A—C2A—C3A	0.2 (3)	C3B—S1B—C1B—C4	−179.6 (2)
Cu1^ii^—N1A—C2A—C3A	−155.55 (18)	C1B—N1B—C2B—C3B	−0.5 (3)
C1A—N1A—C2A—C4A	177.6 (2)	Cu1—N1B—C2B—C3B	177.75 (17)
Cu1^ii^—N1A—C2A—C4A	21.8 (2)	C1B—N1B—C2B—C4B	−178.3 (2)
N1A—C2A—C3A—S1A	0.3 (3)	Cu1—N1B—C2B—C4B	−0.1 (3)
C4A—C2A—C3A—S1A	−176.7 (2)	N1B—C2B—C3B—S1B	0.4 (3)
C1A—S1A—C3A—C2A	−0.6 (2)	C4B—C2B—C3B—S1B	178.0 (2)
C8A—N2A—C4A—C5A	2.3 (3)	C1B—S1B—C3B—C2B	−0.2 (2)
Cu1^ii^—N2A—C4A—C5A	−170.47 (19)	C8B—N2B—C4B—C5B	−1.8 (4)
C8A—N2A—C4A—C2A	−178.9 (2)	Cu1—N2B—C4B—C5B	177.81 (19)
Cu1^ii^—N2A—C4A—C2A	8.3 (3)	C8B—N2B—C4B—C2B	178.2 (2)
C3A—C2A—C4A—N2A	155.6 (2)	Cu1—N2B—C4B—C2B	−2.2 (3)
N1A—C2A—C4A—N2A	−21.4 (3)	C3B—C2B—C4B—N2B	−176.0 (2)
C3A—C2A—C4A—C5A	−25.6 (4)	N1B—C2B—C4B—N2B	1.5 (3)
N1A—C2A—C4A—C5A	157.4 (2)	C3B—C2B—C4B—C5B	4.0 (4)
N2A—C4A—C5A—C6A	−1.4 (4)	N1B—C2B—C4B—C5B	−178.5 (2)
C2A—C4A—C5A—C6A	179.9 (2)	N2B—C4B—C5B—C6B	2.3 (4)
C4A—C5A—C6A—C7A	−0.7 (4)	C2B—C4B—C5B—C6B	−177.7 (2)
C5A—C6A—C7A—C8A	1.8 (4)	C4B—C5B—C6B—C7B	−1.0 (4)
C4A—N2A—C8A—C7A	−1.1 (4)	C5B—C6B—C7B—C8B	−0.6 (4)
Cu1^ii^—N2A—C8A—C7A	170.5 (2)	C4B—N2B—C8B—C7B	0.0 (4)
C6A—C7A—C8A—N2A	−1.0 (4)	Cu1—N2B—C8B—C7B	−179.53 (19)
N2A^i^—Cu1—N1B—C1B	−35.2 (3)	C6B—C7B—C8B—N2B	1.2 (4)

Symmetry codes: (i) −*x*+1, *y*+1/2, −*z*+1/2; (ii) −*x*+1, *y*−1/2, −*z*+1/2.
